# Role of Biomarkers in Periprosthetic Joint Infections

**DOI:** 10.3390/diagnostics12122958

**Published:** 2022-11-25

**Authors:** Serhat Akcaalan, Halil Ibrahim Ozaslan, Ceyhun Caglar, Mehmet Emin Şimşek, Mustafa Citak, Mustafa Akkaya

**Affiliations:** 1 Kırıkkale Yuksek Ihtısas Hospital, Kırıkkale 71300, Turkey; 2 Department of Orthopedics and Traumatology, Faculty of Medicine, Ankara Yildirim Beyazit University, Ankara 06010, Turkey; 3 Department of Orthopedics and Traumatology, Ankara City Hospital, Ankara 06800, Turkey; 4 Department of Orthopedics and Traumatology, Faculty of Medicine, Lokman Hekim University, Ankara 06230, Turkey; 5 Helios ENDO-Klinik Hamburg, 22767 Hamburg, Germany

**Keywords:** periprosthetic joint infection, biomarker, blood, synovial fluid

## Abstract

Periprosthetic joint infection (PJI) is one of the most serious complications after joint arthroplasty. The incidence rate of PJI after total joint replacement is 1–3%. Although there are different guidelines and diagnostic criteria used to diagnose PJI, diagnosing PJI is a highly difficult process for orthopedists. The current Musculoskeletal Infection Society (MSIS) criteria are widely used for the diagnosis of PJI. These criteria include results from blood/synovial fluid tests, physical examination, and histological and microbiological analyses of intra-operative samples. However, there is currently no blood or synovial test that can definitively diagnose PJI. To make a more effective diagnosis of PJI, a large number of studies have explored and continue to investigate biomarkers. This review aims to provide general information about serum and synovial markers used for the diagnosis of PJI that may be used to create a database to guide researchers in new studies.

## 1. Introduction

In recent years, total joint arthroplasty has increased patient quality of life by both relieving pain and increasing mobility [1]. Due to the increase in the average life expectancy, it is thought that there will be a significant increase in the number of total joint arthroplasty operations starting in the 2030s [2]. Periprosthetic joint infection (PJI) is one of the most serious complications after joint arthroplasty [3]. The incidence rate of PJI after total joint replacement is 1–3% [4].

PJI has adverse effects on both the joint and the patient’s quality of life. It causes significant mortality and economic burden [5]. Although there are different guidelines and diagnostic criteria to establish the diagnosis of PJI, diagnosis is a highly difficult process for physicians [3].

For the diagnosis of PJI, the current Musculoskeletal Infection Society (MSIS) criteria are frequently utilized. These criteria include results from blood/synovial fluid tests, physical examination, and histological and microbiological analyses of intra-operative samples [6]. Some criteria in this guideline were updated at the 2018 International Consensus Meeting (ICM) on PJI [7]. However, there is currently no single blood or synovial test that can definitively diagnose PJI.

This review aims to provide general information about serum and synovial markers used for the diagnosis of PJI that may be used to create of a database to help guide researchers in the development of new studies. The goal of the review is to facilitate discussions of possible future developments by interpreting data reported in the current literature.

This study intends to shed light on the function of synovial and serum biomarkers in PJI diagnosis. Serum and synovial fluid biomarkers are shown in Table 1.

## 2. Serum Biomarkers

The use of serum inflammatory parameters for the diagnosis of periprosthetic joint infection is recommended by some researchers [8]. Serum inflammatory parameters are easy to obtain, inexpensive to test, and easy to apply in practice [9]. However, biomarkers in serum are systemic parameters; therefore, they are not specific and do not diagnose PJI alone [10]. Nevertheless, serum biomarkers can be used to aid in the screening and/or diagnosis of PJI [9].

### 2.1. Serum CRP Level and ESR

Serum biomarkers are crucial for the diagnosis of PJI since they involve non-invasive tests. In line with the recommendations of the 2013 International Consensus Meeting on PJI, the serum C-reactive protein (CRP) level and erythrocyte sedimentation rate (ESR) can be used for diagnosis [6]. Since they are included in the diagnostic criteria, these two parameters are the most frequently studied biomarkers in the literature [11]. They are frequently used in the diagnosis of PJI because of their high sensitivity and ease of accessibility. While the sensitivity of ESR is 42–94%, its specificity is 33–87% [11]. While the sensitivity of the CRP level is 74–94%, its specificity varies between 20% and 100% [9]. These parameters are not specific for inflammation of infectious origin because their levels may increase after trauma, autoimmune disease, and surgical procedure [12]. They are commonly used in the diagnosis of PJI because of their high sensitivity and accessibility, but their efficacy when used alone is poor because of their low specificity and lack of increase in some low-virulence bacterial infections [13].

### 2.2. Serum WBC Count

Serum white blood cells (WBC) have a very limited role in the diagnosis of PJI, since a variety of infections can be diagnosed using the serum WBC count [12]. However, due to its ease of availability, the serum WBC count is still used by many surgeons as a guide in the diagnosis of PJI. A prospective study revealed that the sensitivity of the serum WBC level is 21% and its specificity is 94% [14]. There are some inconsistencies in terms of serum WBC counts in studies comparing septic and aseptic revision groups in the literature. Some researchers have reported a higher serum WBC count in the septic group than in the aseptic group [15,16]. Conversely, other researchers have reported no difference in serum WBC values between septic and aseptic groups in their studies [17,18]. Due to the low sensitivity stated in the literature, the serum WBC value may not contribute to the diagnosis of PJI when used alone [9].

### 2.3. Serum IL-6 Level

IL-6 is a cytokine released from fibroblasts, endothelial cells, macrophages, monocytes, and T2 lymphocytes in the presence of bacterial infection/tissue damage [19]. A 2007 study reported that the serum IL-6 level had 95% specificity and 87% sensitivity in the diagnosis of PJI. [16]. A meta-analysis conducted in 2010 showed that the serum IL-6 level was superior to the serum CRP level and ESR in diagnosing PJI, with a sensitivity of 97% and specificity of 91% [20]. Although it has very good sensitivity and specificity, the fact that studies have quite different cut-off values make it difficult to use the serum IL-6 level alone in the diagnosis of PJI [9]. However, its use in combination with the serum CRP value in the diagnosis of PJI has provided excellent results (sensitivity 100%, specificity 99%, accuracy 98%) [19].

### 2.4. Percentage of Serum Neutrophils

As the serum WBC level rises in many infectious conditions, the serum neutrophil percentage may also increase in many cases [21]. This situation explains the emergence of PJI. Easily obtained from routinely requested blood samples in preoperative and post-operative periods, the serum neutrophil percentage is used by clinicians as a screening tool upon suspicion of disease rather than for diagnosis of PJI. The largest study examining the role of the serum neutrophil percentage in PJI diagnosis was published in 2012. This study reported the sensitivity of the serum neutrophil percentage as 53% and its specificity as 75%, when the cut-off value was accepted as 69% [22]. Its low sensitivity and specificity and high values in most infectious conditions prevent the serum neutrophil percentage from being used as a sole diagnostic tool in the diagnosis of PJI.

### 2.5. Serum Procalcitonin Level

The serum procalcitonin (PCT) level was used extensively as a sepsis marker at the beginning of the 2000s [23]. Unless an abnormal situation is encountered, PCT is produced by thyroid gland cells, whereas they are produced by macrophages and liver-derived monocytic cells in infectious conditions [24]. Since a similar situation occurs in PJI, an increased serum PCT level can be used in the diagnosis of PJI. However, there are different data reported for sensitivity and specificity in the literature. A study from 2007 measured the sensitivity of the serum PCT level at 33% and its specificity at 98%, whereas a study from 2020 reported the sensitivity at 40% and specificity at 90% [16,24]. While its low sensitivity makes it difficult to use the serum PCT level alone in the diagnosis of PJI, its sensitivity in diagnosing PJI increases to 80% when combined with the serum CRP level [25]. Therefore, using the serum PCT level alone in the diagnosis of periprosthetic joint infection is not very effective.

### 2.6. Serum Neutrophil Lymphocyte Ratio

The increase in neutrophil count and decrease in lymphocyte count in bacterial infections has made it possible to use the serum neutrophil lymphocyte ratio (NLR) as an infection parameter [26]. Prior to being utilized to diagnose PJI, the serum NLR was used to detect infection during surgical operations across a variety of disciplines [27,28]. Different studies have been conducted to examine the sensitivity and specificity of the serum NLR. These studies evaluated its efficacy in PJI diagnosis by contrasting the serum NLR with CRP and fibrinogen levels. In a study published in 2020, the sensitivity of serum NLR was reported as 63% and its specificity as 73%, indicating that it was less effective than fibrinogen and CRP levels in diagnosing PJI [10]. The low sensitivity and specificity was blamed on the fact that there is no significant inflammatory and immunologic response in low grade infections [29]. Again, for similar reasons, it was stated that the serum NLR may produce false negative results at a rate of 20% [10]. The low sensitivity and specificity of the serum NLR and high rate of false negative results suggest that its use in the diagnosis and screening of PJI will not be effective.

### 2.7. Number of Serum Platelets/Mean Platelet Size

In both inflammatory and infectious conditions, the platelet count (PC) increases as a defense mechanism while the size of the platelets (mPV) decreases due to this numerical increase [30,31,32,33]. In infectious and inflammatory conditions, these changes in opposite directions cause an increase in the PC/mPV ratio. This ratio was first used to diagnose PJI in 2019 and its sensitivity was reported as 48% with a specificity of 81% [32]. In this study, the cut-off value for the PC/mPV value was determined as 31.7, while in a different study, the same value was determined as 29.4. However, these studies obtained similar sensitivity and specificity values [9]. Although the PC/mPV ratio shows good specificity, its low sensitivity indicates that it is not a suitable serum parameter for use alone in diagnosing PJI.

### 2.8. Serum Fibrinogen Level

The serum fibrinogen level is a parameter routinely studied by surgeons to assess coagulation function in the preoperative period [33]. The serum fibrinogen level, like other parameters, has been associated with infection and inflammation [34]. Fibrinogen is synthesized from liver cells and plays an important role in the bleeding cascade [35]. In a meta-analysis published in 2021, the sensitivity and specificity of serum fibrinogen level in diagnosing PJI were reported to be 95% [36]. Compared with the CRP level and ESR, the serum fibrinogen level showed similar results in diagnosing PJI [37]. Despite these positive results in the diagnosis of PJI, the serum fibrinogen level may be affected in different ways in conditions such as lower extremity venous thrombosis, malignancy, autoimmune disease, cerebrovascular disease, and cardiovascular disease [37]. Total joint arthroplasty is mostly performed in elderly patients in whom it is common to encounter these comorbidities. Therefore, despite its good sensitivity, specificity, and cost-effectiveness due to its routine preoperative use, the serum fibrinogen level is not recommended for use alone in the diagnosis of PJI.

### 2.9. Serum D-Dimer Level

D-dimer is a degradation product resulting from fibrin-clot dissolution by plasmin and provides information about the coagulation status [38]. The increase in D-dimer level due to increased fibrinolytic activity because of systemic and local infections explains its use in the diagnosis of PJI [38]. The D-dimer level may be a viable biomarker for the identification of PJI, according to a 2017 study reporting that the blood D-dimer level had higher sensitivity and specificity than the serum CRP level and ESR [39]. Following these positive results, the serum D-dimer level was accepted as a diagnostic criteria at the 2018 International Consensus Meeting on PJI. In a meta-analysis, the sensitivity and specificity of the serum D-dimer level for the diagnosis of PJI were both reported as 95% [36]. Different studies have also demonstrated that the serum D-dimer is an excellent biomarker for the diagnosis of PJI [9,36]. However, despite having high sensitivity and specificity, it was also noted in a different study conducted in 2020 that when used alone, the serum D-dimer level was insufficient to diagnose PJI [40].

### 2.10. Serum Albumin-Globulin Level and Albumin/Globulin Ratio

Albumin and globulin are two important proteins in serum [41]. In diseases in which the inflammatory process is predominant, the albumin level tends to decrease while the globulin level tends to increase [42]. Studies have shown the extent of globulin level and albumin/globulin ratio change in conditions such as infectious conditions, malignancy, and hepatitis [43,44]. For this reason, studies have been conducted exploring their use in the diagnosis of PJI. In a study published in 2022, the sensitivity and specificity of the albumin level, globulin level, and AGR in diagnosing PJI were reported as 75%/69%, 58%/83%, and 65%/86%, respectively [45]. The same study also suggested that AGR may be a promising biomarker for the diagnosis of PJI, particularly given that it produces comparable results to those of the widely used biomarkers ESR and CRP level [45]. Another study published in 2022 also reported that these three biomarkers can be used to diagnose PJI [45]. More studies exploring these parameters are needed since their ease of accessibility will strengthen the ability of clinicians to diagnose PJI.

## 3. Biomarkers in Synovial Fluid

The levels of some biomarkers in serum are not increased in low-grade virulence infections, even though they are included in the ICM diagnostic criteria for the diagnosis of PJI [46]. This has led researchers to find biomarkers that can be detected outside the blood. Consequently, many biomarkers have been identified in synovial fluid.

### 3.1. Synovial Fluid WBC Count and Neutrophil Percentage

The total leukocyte count and neutrophil percentage in synovial fluid are two different parameters frequently used in the diagnosis of PJI [47]. The MSIS criteria for the diagnosis of PJI also include the synovial fluid WBC count. Studies have shown that both the synovial fluid WBC count and neutrophil percentage can be successful diagnostic tools for the diagnosis of PJI [48,49]. In different studies, a range of 1000–5000 cells/μL was determined for the synovial fluid WBC count and the cut-off value was determined as 60–89% for the synovial fluid neutrophil percentage [47]. When 1700 cells/μL was taken as the cut-off value, the sensitivity of the WBC count in synovial fluid was 94% and its specificity was 88%, and when the cut-off value was accepted as 65%, the sensitivity of the neutrophil percentage in synovial fluid was 97% and its specificity was 98% [50]. The existing literature contains various sensitivity and specificity values for various cut-off settings. Although they have advantages in diagnosing PJI, like all synovial fluid examinations, there are some limitations in using the WBC count and neutrophil percentage in synovial fluid [51]. The need for a high-quality synovial fluid sample in order to conduct an effective evaluation is one of the most significant limitations in this respect. Therefore, joint fluid aspiration should be performed under appropriate conditions and by experienced people [50], since blood admixture in the joint fluid makes both quantitative and qualitative examination difficult [50]. An appropriately shaped and high-quality synovial fluid sample can play a key role in diagnosing PJI.

### 3.2. Synovial CRP Level

CRP is an inflammatory marker that is synthesized and secreted by many different cells, such as hepatocyte cells, smooth muscle cells, and endothelial cells, and is frequently used in the diagnosis of infectious diseases [52]. Although this inflammatory marker is frequently used, it is non-specific and has been shown to accumulate intensively in tissue damage/inflammation regions [53]. This characteristic led to the hypothesis that the CRP level in synovial fluid could be utilized to diagnose PJI, and studies have confirmed this hypothesis [54]. The relationship between the serum CRP level and synovial CRP level was demonstrated in different studies and a positive correlation was found between the two. The reason for this finding was stated to be increased vascular and synovial permeability due to the infectious condition [54,55]. In a study published in 2022, which analyzed 621 patients, the sensitivity of the synovial CRP level was determined as 74.2% and its specificity as 98% [56]. The same study also revealed that the synovial CRP level alone was superior to the serum CRP level in diagnosing PJI and showed near perfect accuracy [56]. Even though the synovial CRP level has not been thoroughly investigated and is not currently among the diagnostic criteria for PJI, the synovial CRP level appears to be a promising biomarker for diagnosing the condition. However, further research investigating this biomarker is required.

### 3.3. Synovial IL-6 Level

Since there is an increase in the WBC and PMN% in the synovial fluid due to inflammation, there is bound to be an increase in levels of proinflammatory cytokines, such as interleukins [57]. This led researchers to hypothesize that the synovial IL-6 level could be used in the diagnosis of PJI. The fact that local expression of IL-6 was found to be higher in patients with PJI compared to those with aseptic failure also supported the usefulness of the synovial IL-6 in the diagnosis of PJI [58]. In this context, the synovial IL-6 level has started to be used in the diagnosis of PJI and studies have shown that its sensitivity is 85–100% and its specificity is 62–100% [59]. Despite these successful results, it has been stated that the IL-6 level in synovial fluid is not superior to the WBC count and PMN percentage in synovial tissue in the diagnosis of PJI. Additionally, the synovial IL-6 level does not provide any additional benefit in the diagnosis of PJI [59]. Again, in a meta-analysis published in 2022 supporting these findings, it was stated that the synovial fluid IL-6 level may be more useful as a confirmatory biomarker rather than as a diagnostic biomarker [60].

### 3.4. Synovial Alpha-Defensin Level

Alpha-defensin is an antimicrobial peptide released by activated neutrophils that targets the cell membrane of infective organisms [61]. The sensitivity and specificity of the alpha-defensin level were reported to be 87% and 97%, respectively, in a meta-analysis of research on the subject [62]. Although the alpha-defensin immunoassay has good sensitivity and specificity, comparative studies with routinely used markers are available in the literature because the alpha-defensin immunoassay cannot be routinely performed in most centers and is expensive [63,64]. In patients with total joint arthroplasty requiring two-stage revision, the alpha-defensin immunoassay has been used before re-implantation. It was shown in a different study that testing the synovial alpha-defensin level did not provide any additional benefit [65]. On the contrary, in a large-scale meta-analysis, it was emphasized that the alpha-defensin level was one of the best biomarkers in diagnosing PJI [66]. Despite conflicting views in the literature, incorporating the synovial alpha-defensin test into routine examinations for the diagnosis of PJI appears to be difficult owing to its high cost and challenging accessibility, barring additional studies.

### 3.5. Synovial Procalcitonin Level

Procalcitonin, a precursor of calcitonin, is a protein molecule produced by different cell groups of the thyroid gland and is found at very low levels in serum [67]. Like other markers mentioned in this review, the procalcitonin level increases after bacterial infection [68]. However, a meta-analysis reported that the sensitivity and specificity of the serum PCT level failed to diagnose PJI [69]. For this reason, there have been a limited number of studies examining the PCT levels in synovial fluid to provide a more sensitive and specific diagnostic marker for PJI [70,71]. These studies have shown that the diagnostic accuracy of the synovial PCT level is better than that of the serum PCT level, but both PCT levels do not have sufficient potency to diagnose PJI [71,72].

### 3.6. Synovial Calprotectin Level

Although calprotectin is known as a cystic fibrosis antigen, it is a protein complex that is part of the inflammatory response [73]. This protein complex is secreted by neutrophils and involved in leukocyte migration and stimulation [73]. Even though there are few studies on the use of the synovial calprotectin level in the diagnosis of PJI, a meta-analysis found that this biomarker had a specificity of 84–99% and sensitivity of 84–98% in the diagnosis of PJI [73].In light of these data, it has been stated that the synovial calprotectin level can be used as a reliable biomarker in the diagnosis of PJI and it has been argued that it may be preferred owing to its low cost [74]. These promising developments have been supported by the findings of another recent meta-analysis and study [75,76]. It is obvious that the synovial calprotectin level, a promising agent in the diagnosis of PJI, will increasingly occupy a place in daily practice in the future.

## 4. Discussion

PJI is an important complication of total joint arthroplasty that causes both economic and medical problems. This complication, which affects approximately 1–2% of patients undergoing joint arthroplasty, may lead to revision surgeries and long-term use of systemic antibiotics [5]. Considering the negative effects caused by PJI, early diagnosis and treatment are of great importance [7]. Nevertheless, there is no gold standard method for diagnosing PJI; therefore, there are many studies on this subject in the literature [77]. Although many diagnostic tests are available, none of them can be used alone to make a diagnosis [78]. For this reason, PJI is diagnosed by evaluating together clinical findings, microbiological and histological analyses, serum/synovial fluid examinations, and intraoperative findings [79,80].

Many biomarkers are used and studied in the diagnosis of PJI [9]. Generally, biomarkers are investigated in serum and synovial fluid. However, no single biomarker can diagnose PJI. Serum biomarkers are often preferred because they are inexpensive to test and easy to apply in practice [9]. However, these biomarkers are not specific as they are derived from systemic circulation [9]. Levels of serum biomarkers may also increase in conditions such as autoimmune diseases and after trauma, apart from PJI [12]. In addition to these systemic disadvantages, there may not be an increase in levels of some serum biomarkers, especially in cases of infection with localized and low-virulence pathogens [13]. Due to these disadvantages, the use of synovial biomarkers has emerged in order to provide a local and more specific examination [46]. However, testing of synovial markers is more expensive, difficult to apply in practice, and can yield false positive results in cases such as local soft tissue reactions, hematoma, acute inflammatory arthritis, and crystal arthropathy, which prevents their use alone in the diagnosis of PJI [80]. The diagnostic criteria of PJI are determined by the Musculoskeletal Infection Society and updated from time to time with clinical findings, serum and synovial biomarkers, and histological and microbiological examinations determined by a scoring system [7].

The serum and synovial markers described under separate headings in this review are not the gold standard for diagnosing PJI. Numerous and promising studies are ongoing to find a singular diagnostic tool capable of diagnosing PJI [81]. Among these, it is important to identify genetic polymorphisms that predispose patients to PJI. In case of infection, inflammatory cytokines are released both locally and systemically [82]. Changes in genes encoding many of the proteins that control the release of these cytokines may be susceptible to PJI [83,84]. In this context, many single nucleotide polymorphisms have been identified that are associated with predisposition to PJI [85,86]. In order for these factors to be used more effectively in daily orthopedic practice, studies involving larger populations are needed. Conducting studies involving large populations may provide an opportunity to create biobanks using artificial intelligence that will facilitate personalized, patient-specific treatment and development of new diagnostic approaches [87]. These studies may help provide patient-specific care and identify a gold standard diagnostic tool for PJI.

## 5. Conclusions

The diagnosis of PJI is a highly complex and challenging process. Although numerous studies investigating biomarkers have been completed and are still being conducted in attempt to improve the accuracy of diagnosis, the criteria established by the International Consensus Meeting on PJI are still in use today. To date, the optimal diagnostic test with 100% accuracy is still missing. Further research, especially using gene technology, is needed to find the optimal test.

## Figures and Tables

**Table 1:** Serum and Synovial Biomarkers in Periprosthetic Joint Infections.

Biomarkers in Periprosthetic Joint Infections			
Serum Biomarkers		Synovial Biomarkers	
Serum CRP Level	Serum Procalcitonin Level	Synovial Fluid WBC Count	Synovial Calprotectin Level
ESR	Serum Neutrophil/Lymphocyte Ratio	Synovial Fluid Neutrophil Percentage	
Serum WBC Count	Number of Serum Platelets/Mean Platelet Size	Synovial CRP	
Serum IL-6 Level	Serum Fibrinogen Level	Synovial IL-6 Level	
Percentage of Serum Neutrophils	Serum D-Dimer Level	Synovial Alpha-Defensin Level	
Serum Procalcitonin Level	Serum Albumin-Globulin Level and Albumin/Globulin Ratio	Synovial Procalcitonin Level	
